# Virtual Nursing and the Future of Workforce Sustainability

**DOI:** 10.1001/jamanetworkopen.2025.45603

**Published:** 2025-12-01

**Authors:** Saif Khairat, Bonnie Clipper

**Affiliations:** University of North Carolina at Chapel Hill; Innovation Advantage, Bastrop, Texas

Severe nursing shortages are straining health systems in the US and globally. The workforce crisis remains among the most acute in modern US history. Burnout, turnover, and retirements accelerated by the COVID-19 pandemic have left hospitals struggling to deliver safe care with fewer registered nurses at the bedside.^1^ In this context, virtual nursing, an emerging extension of telehealth that uses remote technology to provide safe, high-quality care through communication and collaboration, has emerged as a strategy to support bedside staff and maintain care quality.^2^ However, a central question remains: do bedside nurses actually feel that virtual nursing helps?

In this study, Muir and colleagues^3^ surveyed nearly 900 bedside nurses across 10 states whose hospitals had implemented virtual nursing. They found that virtual nurses most often assisted with observation, admissions, discharges, and patient education. More than half of respondents reported no change in workload, over half perceived some improvement in quality of care, and a minority experienced relief, while a few described a large benefit. Open-ended responses reflected both advantages, such as an extra set of eyes and help with routine tasks, and drawbacks, including duplication, delays, and patient skepticism.

The study did not describe how virtual nurses were integrated into hospital systems, such as whether they documented directly in the electronic health record (EHR) or required bedside staff to re-enter information. It also did not indicate whether virtual nurses were employed internally or supplied through third-party vendors. Both factors could plausibly shape how bedside staff experienced virtual nursing. For instance, without seamless documentation and escalation privileges, virtual nurses may introduce duplication rather than relief. When remote colleagues are viewed as external rather than part of the team, trust and accountability may be harder to establish.

As with all survey-based studies, the results capture perception rather than causal impact on outcomes. Yet in practice, perception is an essential factor. Adoption of emerging technologies depends on whether frontline clinicians feel supported. The study findings indicated that many nurses reported neutral effects on workload, with only a small minority describing substantial benefits, underscoring that virtual nursing’s effects are not uniform and likely depend on local implementation and environment.

At the same time, health systems should avoid shifting burnout from bedside nurses to virtual nurses. Continuous screen time, as demonstrated in a 2025 virtual nursing study,^4^ creates occupational risks for virtual nurses and should be mitigated through workforce planning and workflow design that incorporates regular breaks.

This study also emphasized the importance of trust, both among staff members and between patients and staff. Bedside nurses described patient skepticism and requests to “hear it again from my nurse in the room.” Protecting the nurse–patient relationship requires introducing virtual nurses as augmentation rather than substitution, because patient acceptance often mirrors whether bedside staff perceive remote colleagues as reliable partners.^5^ Equity considerations also matter, as virtual nursing may extend expertise to rural or underresourced hospitals but depends on reliable connectivity and infrastructure that remain unevenly distributed.

Experiences with prior technological shifts, notably EHRs and telehealth, offer lessons for implementing virtual nursing. Lessons from these experiences should inform implementation strategies of virtual nursing. The adoption of EHRs underscored that technology without workflow redesign and user-centered design risks adding burden; virtual nursing must be paired with practice transformation and change management. Lessons from telehealth have demonstrated that integrating it into existing health systems, rather than operating as a standalone service, yields improved outcomes and a better patient experience. Similarly, virtual nursing should be viewed as a complement to safe baseline staffing rather than a substitute. Future models may also need to consider how virtual nursing interfaces with physicians and other team members to avoid duplication and fragmentation of care. Virtual nurses appear more effective for discrete tasks such as admissions and discharges, where responsibilities can be clearly delineated. The degree to which virtual nurses can document directly in the EHR, escalate issues in a clinical setting, and hand off tasks with clarity may help explain whether bedside nurses experience relief or additional burden.^6^

Virtual nursing must also be considered within policy frameworks that govern the workforce, licensure, financing, and the integration of technology. In the US, questions remain about whether virtual nurses count toward staffing ratios, how Title VIII Nursing Workforce Development programs support hybrid roles, and how the Nurse Licensure Compact addresses scope of practice across state borders.^7,8^

Globally, the World Health Organization *State of the World’s Nursing* report emphasizes redistribution and retention strategies that align with virtual nursing, while cross-border practice raises questions of accountability and liability.^9^ In low- and middle-income countries, virtual nursing may also help mitigate migration-driven shortages; however, feasibility will depend on the availability of digital infrastructure and local workforce policies. Financing frameworks in the US and internationally have yet to explicitly address virtual nursing, despite payment programs increasingly tying reimbursement to outcomes such as readmissions and length of stay.^10^ Robust economic evaluations will also be necessary to assess whether the costs of technology and staffing yield sufficient returns in terms of quality, safety, and efficiency. Clarifying these policy domains will be essential if virtual nursing is to evolve from pilots into a sustainable component of health systems worldwide.

Taken together, these domains underscore that virtual nursing should be regarded not as a temporary fix but as an emerging model of nursing delivery. Its future will depend on regulatory recognition, integration into staffing and quality frameworks, and adaptation to diverse contexts. Muir et al^3^ provide a critical starting point by documenting how frontline nurses viewed its early impact. The next step is to ensure that research and policies keep pace, so that virtual nursing can evolve from fragmented pilots into a sustainable, scalable, trusted, and effective component of health systems worldwide. As virtual nursing programs mature, integration with emerging tools, such as artificial intelligence, may expand opportunities for decision support and efficiency. However, these applications also raise new questions for research and policy.

Realizing the potential of virtual nursing will require answers to several key questions. Whether virtual nursing reduces burnout and improves retention will determine its contribution to alleviating staffing shortages. Its ability to fill workforce gaps, rather than merely redistribute tasks, will shape its value for health systems. Just as important is whether efficiency gains such as shorter stays, fewer readmissions, and safer discharges outweigh the costs of technology and implementation.

Developing consistent evaluation frameworks and metrics will also be necessary to effectively implement and sustain virtual nursing programs, measure outcomes, and guide policy decisions. Future research should examine how employment models, program maturity, and specific use cases influence these outcomes. These are priority areas that policy and research must confront if virtual nursing is to fulfill its promise as part of the future of health care delivery.
